# Glycosylation‐Associated Macrophage Signatures Define a Diagnostic Model for Diabetic Retinopathy via Single‐Cell Analysis

**DOI:** 10.1155/joph/7199483

**Published:** 2026-08-27

**Authors:** Zhujuan Pan, Shaobo Zhou, Wenjuan Qi, Jing Wang, Yan Luo, Feihong Fan

**Affiliations:** ^1^ Key Laboratory of Biological Targeting Diagnosis, Therapy and Rehabilitation of Guangdong Higher Education Institutes, Ophthalmology Department, The Fifth Affiliated Hospital, Guangzhou Medical University, Guangzhou, China, gzhmc.edu.cn

**Keywords:** diabetic retinopathy, diagnostic model, glycosylation, machine learning, macrophage

## Abstract

**Purpose:**

Diabetic retinopathy (DR) remains a leading cause of vision loss, with macrophages and glycosylation dysregulation implicated in DR pathogenesis. However, the potential as diagnostic biomarkers has been rarely investigated.

**Methods:**

We integrated single‐cell RNA sequencing (scRNA‐seq) datasets to profile DR cell landscapes. The immune cell heterogeneity was dissected, and glycosylation‐related transcriptional programs were delineated in DR. Machine learning–based diagnostic modeling was also conducted to identify macrophage differentiation–related glycosylation genes (MDRGGs).

**Results:**

Macrophages exhibited elevated abundance in proliferative DR and showed intense interactions with other monocytes. Endothelial cells were subdivided into four subtypes, with Endo_KCNQ3 representing a dominant proliferative and highly glycosylated phenotype. Monocytes were clustered into three subtypes; Mono_RGS1 emerged as a transitional phenotype in the monocyte‐to‐macrophage trajectory. Macro_MIR181A1HG was identified as a proliferative and glycosylation‐active macrophage subset. A total of 100 MDRGGs were identified. Among them, seven hub genes (AKAP13, SRGAP2, AFF1, ARHGAP24, RNF149, PTK2B, ATP1B3) were incorporated into a diagnostic model. The model achieved high predictive accuracy in both training and external validation cohorts (AUC > 0.85) and was further validated via nomogram and decision curve analyses.

**Conclusion:**

Glycosylation is closely associated with macrophage heterogeneity in DR. A seven‐gene MDRGG‐based diagnostic model demonstrated robust diagnostic performance in DR.

## 1. Introduction

Diabetic retinopathy (DR) represents a chronic and irreversible eye disease, widely recognized as the leading contributor to visual impairment and blindness in individuals of working age [1]. As one of the most common complications associated with diabetes mellitus (DM), DR manifests with an incidence rate that approximates 20%–30% of all DM‐related complications [2]. Despite improvements in clinical management, limitations in early and precise diagnostic strategies hinder timely intervention for DR. Among the numerous pathological processes implicated in DR, chronic inflammation and immune dysregulation, particularly those involving macrophage activation [3], have been increasingly recognized as central contributors to retinal damage and neovascularization. However, whether macrophage‐related molecular signatures could serve as diagnostic biomarkers for DR has not yet been systematically reported.

O‐GlcNAcylation, an essential post‐translational modification of proteins, exerts a significant influence on cellular signaling pathways [4]. O‐GlcNAcylation is stringently regulated by ambient glucose levels [5, 6]. Aberrant glycosylation patterns have been observed in diabetic microenvironments and are closely linked to inflammation, angiogenesis, and tissue remodeling, all of which are central to DR pathogenesis. GnT‐V‐mediated aberrant N‐glycosylation of TIMP‐1 exacerbates angiogenesis in retinal microvascular endothelial cells and induces the cell injury of ARPE‐19 cells in DR [7]. Protein O‐GlcNAcylation interacts with the hippo signaling pathway to activate proangiogenic and glucose metabolism‐related transcriptional programs, thereby contributing to vascular dysfunction in DR [8]. Furthermore, recent studies highlight that RNA N‐glycosylation plays a critical role in modulating immune cell functions, including enabling immune evasion and maintaining homeostatic efferocytosis [9]. Despite these mechanistic insights, studies exploring glycosylation as a diagnostic biomarker in DR remain scarce, and the utility in delineating cellular heterogeneity and disease progression is poorly understood. Therefore, elucidating the diagnostic potential of glycosylation‐related signatures in DR may provide novel insights into the pathogenesis and clinical stratification of DR.

Recent advances in single‐cell technologies have revealed that macrophages, as key innate immune effectors, exhibit substantial heterogeneity and functional plasticity in DR [10]. Beyond their classical pro‐ and anti‐inflammatory polarization, emerging studies have highlighted the existence of distinct macrophage subpopulations that dynamically participate in retinal homeostasis, inflammation, and neovascularization [11–13]. Despite these findings, a comprehensive understanding of macrophage differentiation trajectories, particularly under diabetic conditions, is lacking. Moreover, glycosylation has garnered substantial attention as a regulatory mechanism for orchestrating macrophage differentiation [14, 15]. However, the regulatory role of glycosylation in shaping macrophage heterogeneity and functional states in DR remains largely unexplored. Notably, investigations into the glycosylation of macrophages as potential diagnostic biomarkers in DR are very limited, underscoring the need to characterize glycosylation‐related macrophage signatures to improve disease diagnosis and stratification.

To characterize macrophage heterogeneity driven by glycosylation in the DR microenvironment, we employed integrated single‐cell RNA sequencing (scRNA‐seq) datasets to generate a detailed cellular landscape of DR. Based on comprehensive bioinformatics analyses, the glycosylation‐modified genes associated with macrophage polarization in DR were elucidated. Furthermore, a diagnostic model for DR was constructed by machine learning techniques. This research provides novel insights into the pathogenesis of DR and offers valuable references for subsequent clinical diagnostics of the condition.

## 2. Methods

### 2.1. Data Acquisition

ScRNA‐seq datasets of DR were obtained from the Gene Expression Omnibus (GEO) database (https://www.ncbi.nlm.nih.gov/geo/), including GSE245561 (four preretinal fibrovascular membrane [FVM) samples from proliferative diabetic retinopathy [PDR] patients) [16], GSE165784 (five FVM samples from PDR patients) [17], and GSE248284 (three peripheral blood mononuclear cells [PBMCs] from DR samples) [18]. Among these, a total of nine PDR samples were selected for single‐cell data analysis. Bulk RNA sequencing (bulk RNA‐seq) datasets of DR were downloaded from GEO, including GSE102485 (comprising 22 DR and 3 control samples) [19] and GSE160306 (consisting of 20 DR and 20 control samples) [20]. For the construction and analysis of the diagnostic model, a total of 42 DR and 23 control samples were selected from these bulk RNA‐seq cohorts.

### 2.2. ScRNA‐seq Analysis

After importing the gene count matrix into the “seurat” package (Version 5.0.3), quality control was performed to remove low‐quality cells and genes. Genes expressed in fewer than 3 cells were excluded. Cells with fewer than 200 or more than 9000 detected genes, as well as those with total UMI counts (Tag counts) less than 500 or greater than 1,00,000, were removed to eliminate potential empty droplets or doublets. These thresholds were determined based on the distribution of quality‐control metrics and prior scRNA‐seq studies. In addition, the mitochondrial gene content was then calculated for each cell, and cells with mitochondrial gene expression exceeding 25% were filtered out, based on the observed distribution of quality‐control metrics in our dataset, as a high mitochondrial content is generally indicative of stressed or low‐quality cells in scRNA‐seq data. The remaining cellular data were then normalized using the NormalizeData function. Principal component analysis (PCA) was performed on the processed data. Subsequently, batch effect correction and dimensionality reduction were conducted using the Harmony algorithm [21] based on the PCA embeddings.

Uniform manifold approximation and projection (UMAP) was applied to display the data after dimensionality reduction. Cell clustering was performed via the k‐nearest neighbor (k‐NN) algorithm to partition cells into distinct subpopulations. Cell‐type annotation was subsequently conducted using the BlueprintEncodeData reference dataset from the “singleR” package (Version 1.6.1) and “celldex” package (Version 1.2.0). Annotation results were verified and refined using established cell marker genes. Differentially expressed genes (DEGs) were identified through the FindAllMarkers approach. Cell–cell communication analysis was performed using the “cellchat” package (Version 1.6.1). Pseudotime trajectory analysis used “monocle2” package (Version 2.26.0). Unless otherwise indicated, all scRNA‐seq analyses were conducted using the integrated PDR FVM datasets GSE245561 and GSE165784. Only the pseudotime analysis included an additional PBMC dataset (GSE248284) to investigate the monocyte‐to‐macrophage differentiation trajectory.

### 2.3. Glycosylation Score

Glycosylation‐related gene set enrichment scores were computed for individual cells using single sample gene set enrichment analysis (ssGSEA) implemented in the “GSVA” package (Version 1.46.0) to evaluate the glycosylation score. Based on the median glycosylation score, cells were categorized into high‐ and low‐score groups. DEGs between high‐ and low‐glycosylation score groups were determined based on the criteria of |avg_log_2_FC| > 1 and *p* < 0.05. Violin plots visualizing the distributional differences in glycosylation scores between these stratified groups were generated using the “ggplot2” package (Version 3.4.2).

### 2.4. GSVA

KEGG pathway enrichment scores were computed for all identified cell subpopulations using the GSVA package (Version 1.46.0) with gene sets retrieved from the “msigdbr” package (Version 7.5.1). The resulting pathway enrichment profiles were visualized through hierarchically clustered heatmaps generated with the “heatmap” package (Version 1.0.12).

### 2.5. Weighted Gene Coexpression Network Analysis (WGCNA)

Gene coexpression networks were constructed in macrophage populations using the “hdWGCNA” package (Version 0.4.00) to identify biologically relevant modules. Correlational analyses were then performed between module and both glycosylation score and macrophage activation stages.

### 2.6. Machine Learning Analysis

To identify robust diagnostic biomarkers of DR based on macrophage differentiation–related glycosylation genes (MDRGGs), we implemented a robust machine learning framework integrating pairwise combinations of 11 distinct algorithms. Gene expression data from GSE160306 served as the training cohort, while GSE102485 was used as an independent validation dataset. The following machine learning algorithms were employed: generalized linear model boosting (glmBoost), elastic net (Enet), random forest (RF), stepwise generalized linear model (Stepglm), extreme gradient boosting (XGBoost), support vector machine (SVM), ridge regression (Ridge), least absolute shrinkage and selection operator (LASSO), partial least squares regression for GLM (plsRglm), naive Bayes classifier (NaiveBayes), and linear discriminant analysis (LDA). All possible pairwise combinations of these models were constructed to enhance prediction robustness. For each model combination, we calculated AUC values in both the training and validation datasets. The model pair with the highest average AUC values across the two datasets was selected as the optimal diagnostic model. Genes consistently prioritized by the optimal model were designated as hub diagnostic biomarkers of DR. These biomarkers were further subjected to receiver operating characteristic (ROC) analysis to validate their diagnostic accuracy by R package “pROC” (Version 1.18.5).

### 2.7. Clinical Nomogram Analysis

Based on the hub genes, the GSE102485 dataset was selected to construct a clinical nomogram for DR using R package “regplot” (Version 1.1). We employed hub genes to yield a predicted score for each patient. Characteristics with a *p* value of < 0.05 in the regression analysis were selected for constructing a nomogram for DR. To assess the precision of nomogram predictions, the calibration curve analysis (R package “rms,” Version 8.0.0) and decision curve analysis (DCA)analyses (R package “dcurves,” Version 0.5.0.9000) were conducted in the GSE102485 dataset.

## 3. Results

### 3.1. Highly Heterogeneous Macrophages Exhibited Relatively Elevated Abundance in PDR

**FIGURE 1:**
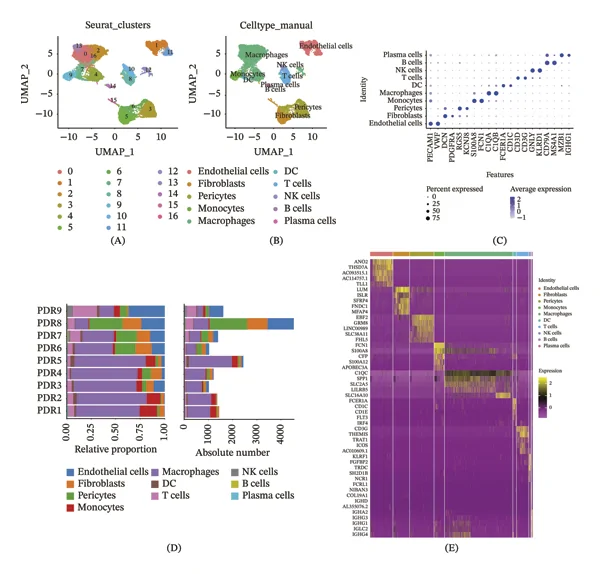
Highly heterogeneous macrophages exhibited relatively elevated abundance in PDR. (A) UMAP visualization of 17 single‐cell clustering. (B) UMAP plot showing manual annotation of cell types. (C) Dot plot depicting the expression of cell subtype–specific marker genes across different cell identities. (D) Stacked bar plots illustrate the relative proportion (left) and absolute number (right) of each cell subtype across nine PDR samples. (E) Heatmap of gene expression patterns for 10 cell subtypes.

To elucidate the single‐cell transcriptome profiles of DR, we integrated two scRNA‐seq datasets GSE245561 and GSE165784, containing nine PDR samples. After stringent quality control, a total of 16,064 cells were divided into 17 clusters (Figure 1A). The quality‐control assessment demonstrated good data reliability, with a strong positive correlation between nCount_RNA and nFeature_RNA (*r* = 0.86) and a weak negative correlation between nCount_RNA and percent mitochondrial gene expression (*r* = −0.20), indicating effective removal of low‐quality and stressed cells (Figure S1). Then, 10 cell subtypes were identified by specific markers (Figure 1B,C). These cell subtypes included endothelial cells (PECAM1, VWF), fibroblasts (DCN, PDGFRA), pericytes (RGS5, KCNJ8), monocytes (S100A8, FCN1), macrophages (C1QA, C1QB), dendritic cells (FCER1A, CD1C), T cells (CD3D, CD3G), nature kill cells (GNLY, KLRD1), B cells (CD79A, MS4A1), and plasma cells (MZB1, IGHG1). The distribution of cellular populations in PDR exhibits significant interindividual variability. ScRNA‐seq data revealed that macrophages constituted a predominant cell population in samples PDR1, PDR2, PDR3, and PDR5, accounting for 64%–72% of the total cells (Figure 1D). Conversely, in samples PDR6, PDR7, PDR8, and PDR9, the proportion of macrophages was markedly reduced (14%–38%), while the proportion of pericytes increased substantially (20%–34%), (Figure 1D), implying the presence of anomalous angiogenic processes in these samples [22]. We further investigated the DEGs within each cellular subtype and identified that C1QC, SPP1, ALC2A5, LILRB5, and SLC16A10 exhibited upregulation specifically in macrophages (Figure 1E). In conclusion, these results highlight the pronounced heterogeneity of macrophage populations within PDR samples.

### 3.2. Enhanced Intercellular Crosstalk of Macrophages and Monocytes in DR

**FIGURE 2:**
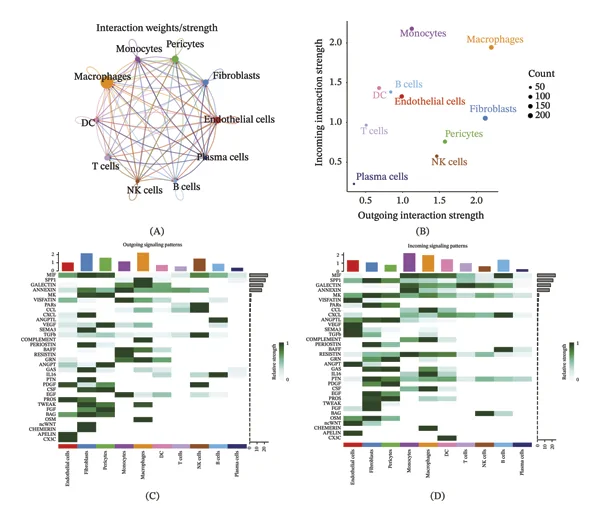
Enhanced intercellular crosstalk of macrophages and monocytes in DR. (A) Cell–cell interaction network depicting interaction weights among different cell subtypes. (B) Scatter plot of outgoing and incoming interaction strengths for different cell subtypes. (C) Heatmap of outgoing signaling patterns across cell subtypes. (D) Heatmap of incoming signaling patterns across cell types.

To investigate the cellular interaction landscape in DR, cell chat analysis was conducted. The circos plot provides a descriptive overview of inferred interaction intensity and frequency among FVM cell populations, with apparent interactions observed between macrophages and endothelial cells (Figure 2A). Macrophages were identified as the predominant signal‐sending cell subtype in DR via the SPP1, GALECTIN, COMPLEMENT, GRN, and OSM signaling pathways (Figure 2B,C). Macrophages were identified as a cell subtype with the higher incoming interaction strength than other subtypes (Figure 2B, C). In contrast, monocytes were the predominant signal‐receiving cell subtype, receiving signals through the MIF, ANNEXIN, and RESISTIN signaling pathways (Figure 2B,D). Importantly, pericytes also demonstrated substantial outgoing signaling activity, highlighting their active contribution to vascular communication in DR (Figure 2B). Pericytes primarily transmitted signals through key pathways, including MK (midkine), VEGF, ANGPT, CSF, and FGF (Figure 2B,C). Conclusively, these findings indicate that both macrophages and monocytes exhibit markedly intensified interactions with diverse FVM cell populations in DR.

### 3.3. Endo_KCNQ3 Defines a Dominant Proliferative Endothelial Phenotype in PDR

Given the pivotal role of endothelial dysfunction in the pathogenesis of DR, subcluster analysis was conducted to investigate the transcriptional heterogeneity of endothelial cells. Endothelial cells were segregated into four distinct clusters (Figure 3A). Subsequent annotations based on identified markers revealed four endothelial subtypes: Endo_KCNQ3, Endo_LINC0047, Endo_PRL39, and Endo_MT‐RNR (Figure 3B). Marker gene expression profiles for each subtype are displayed in Figure 3C. Among these, Endo_KCNQ3 comprised the largest proportion of endothelial cells (Figure 3D). GSVA analysis revealed distinct functional programs among the four endothelial subtypes (Figure 3E). Endo_KCNQ3 was strongly enriched in pathways associated with fibrosis commitment, such as hedgehog signaling, WNT beta catenin signaling, TGF beta signaling, and mitotic spindle. Given that fibrotic remodeling is a key pathological event in DR [23], the involvement of Endo_KCNQ3 highlights its potential role in promoting fibrotic progression. Endo_PRL39 displayed enrichment in signaling networks related to cellular plasticity, including myogenesis, epithelial mesenchymal transition, apical surface, angiogenesis, and reactive oxygen species pathway. In contrast, Endo_MT‐RNR was characterized by the upregulation of immune and inflammatory response pathways (inflammatory response, complement, interferon alpha response, and interferon gamma response). Collectively, these findings suggest that distinct endothelial subpopulations exert heterogeneous yet complementary roles in DR progression, involving fibrotic remodeling, inflammatory activation, and phenotypic plasticity. Differential expressions of immune‐related regulatory molecules highlighted distinct immunoregulatory states across the subtypes (Figure 3F). Notably, macrophage‐associated signaling genes, including CD40 and PDGFB, were predominantly enriched in Endo_KCNQ3, suggesting potential involvement of endothelial–macrophage crosstalk in the DR microenvironment (Figure 3G). Moreover, Endo_KCNQ3 exhibited the highest glycosylation score among the subtypes, implying metabolic reprogramming under hyperglycemic conditions (Figure 3H). Pseudotime trajectory analysis further revealed that Endo_KCNQ3 was predominantly enriched in the intermediate‐to‐late stages of endothelial differentiation (Figure 3I). Taken together, Endo_KCNQ3 emerges as the dominant endothelial subtype in DR, characterized by a combined signature of profibrotic signaling, immunomodulatory potential, and metabolic reprogramming.

FIGURE 3Endo_KCNQ3 defines a dominant proliferative endothelial phenotype in PDR. (A) UMAP visualization of endothelial cells showing segregation into four distinct clusters. (B) Annotation of the four clusters into four endothelial subtypes. (C) Heatmap illustrates the representative marker genes identified for each endothelial subtype. (D) Bar plot demonstrates the proportion of each endothelial subtype in PRD. (E) GSVA revealing distinct pathway activity across four endothelial subtypes. (F) Violin plots displaying the expression levels of immune‐related regulatory genes across four endothelial subtypes. (G) Expression profiles of macrophage‐associated signaling genes across four endothelial subsets. (H) The levels of glycosylation scores across four endothelial subsets. (I) Pseudotime trajectory analysis depicting the differentiation trajectory of four endothelial subtypes in PDR.

**Figure:**
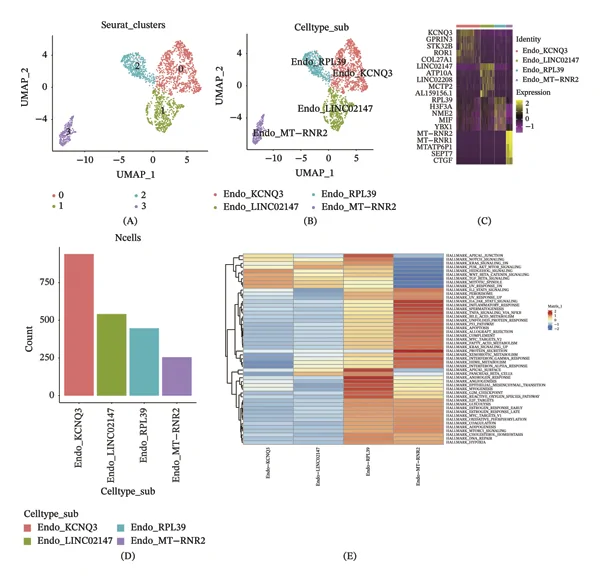

**Figure:**
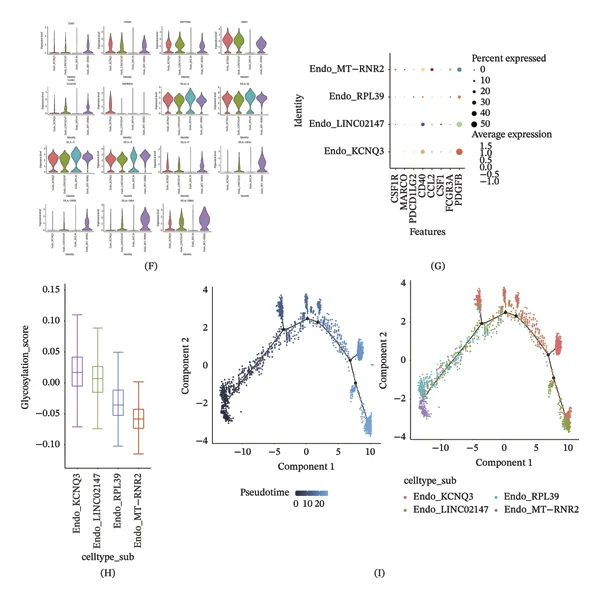

### 3.4. Heterogeneous Functional States of Monocytes in PDR

Given that circulating monocytes give rise to tissue macrophages that drive chronic inflammation and neovascularization in DR, we subclustered monocytes to map the transcriptional heterogeneity in DR. Monocytes were classified into three clusters, corresponding to three transcriptionally distinct subtypes: Mono_S100A9, Mono_RGS1, and Mono_MICOS10 (Figure 4A,B). Proportion analysis revealed that Mono_S100A9 constituted the dominant population, accounting for 48.43% of all monocytes and Mono_RGS1 represented 31.89% (Figure 4C). Subtype‐specific marker genes were identified for each population, including Mono_S100A9 (S100A9, S100A8, S100A12, SELL, and NCF1C), Mono_RGS1 (RGS1, TREM2, C3, MRC1, and APOE), and Mono_MICOS10 (MICOS10, SEPTIN7, TOMM6, TUT7, and LINC00278) (Figure 4D). Analysis of immune‐related regulatory gene expression revealed differential immunomodulatory profiles among the subtypes (Figure 4E). GSVA further delineated the functional heterogeneity among the three monocyte subtypes (Figure 4F). Mono_S100A9 was significantly enriched in pathways related to cellular stress response, such as UV response and reactive oxygen species pathway. Mono_RGS1 showed preferential activation of inflammation‐ and immune interaction‐related signaling pathways, including inflammation responses, interferon gamma responses, and complement. Mono_MICOS10 was enriched in pathways associated with cell development and differentiation (WNT beta signaling, notch signaling, hedgehog signaling, and Pi3k Akt mTOR signaling), reflecting a metabolically active. Notably, Mono_RGS1 exhibited the highest TAM‐related signature score (Figure 4G), suggesting enhanced macrophage‐associated immune regulatory features. In contrast, Mono_MICOS10 displayed the highest glycosylation score, reflecting metabolic adaptation during advanced cellular transitions (Figure 4H). Pseudotime trajectory analysis placed Mono_S100A9 at the initial stage, Mono_RGS1 at an intermediate state, and Mono_MICOS10 at the terminal stage of differentiation (Figure 4I). In summary, three monocyte subtypes display distinct functional states in DR, outlining a dynamic trajectory of monocyte progression in DR.

FIGURE 4Heterogeneous functional states of monocytes in PDR. (A) UMAP visualization shows three transcriptionally distinct monocyte clusters. (B) Annotation of clusters into three monocyte subtypes. (C) Pie plot demonstrates the proportion of each monocyte subtypes in PRD. (D) Expression of representative marker genes across three monocyte subtypes. (E) Expression profiles of immune‐related regulatory genes among three monocyte subtypes. (F) GSVA analysis revealing distinct functional signatures across three monocyte subtypes. (G) Expression profiles of TAM‐related signature score across three monocyte subtypes. (H) The levels of glycosylation scores across three monocyte subtypes. (I) Pseudotime trajectory analysis depicting the differentiation trajectory of three monocyte subtypes in PDR.

**Figure:**
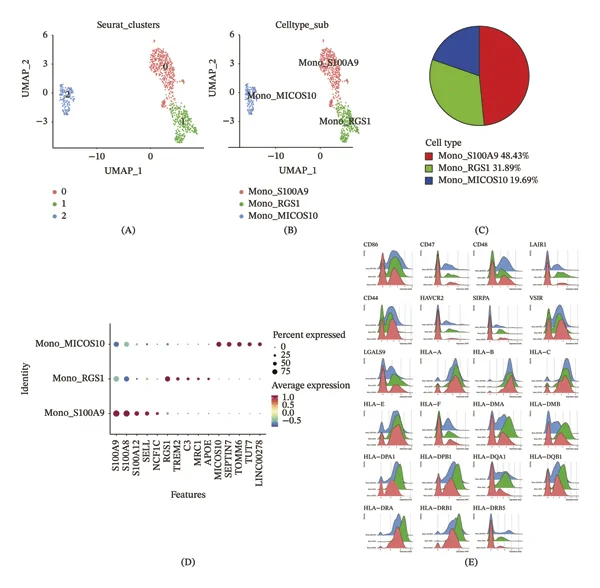

**Figure:**
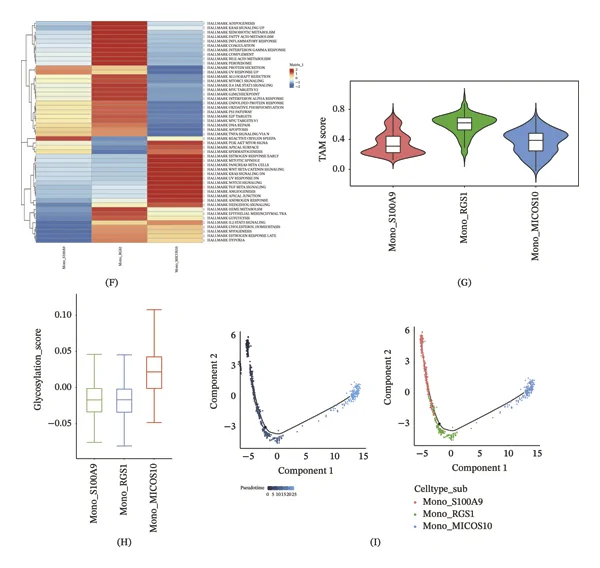

### 3.5. Macro_MIR181A1HG Represents a Proliferative and Glycosylation‐Active Macrophage Subtype in PDR

As macrophages constitute the dominant immune cell population in PDR, detailed subclustering analysis was conducted to characterize the heterogeneity within the DR microenvironment. A total of five clusters were identified as five macrophage subtypes in PDR (Figure 5A–C). These macrophage subtypes included Macro_P2RY13, Macro_MIR181A1HG, Macro_MALAT1, Macro_GCHFR, and Macro_CD163L1 (Figure 5D). The relative abundance of these subtypes varied considerably across individual PDR samples, highlighting pronounced intersample heterogeneity in DR (Figure 5D). GSVA analysis was employed to investigate the biological function of distinct macrophage subtypes in DR. The heatmap revealed that the Macro_MIR181A1HG was associated with cell proliferation signaling pathways, including G2M checkpoint, KRAS signaling dn, and PI3K AKT mTOR signaling (Figure 5E). Additionally, Macro_P2RY13 regulated signaling pathways related to immune response, such as interferon *α*/*γ* response, IL6‐JAK‐STAT3 signaling, allograft rejection, and apoptosis (Figure 5E). Meanwhile, the Macro_GCHFR was implicated in cellular stress responses, including hypoxia and reactive oxygen species pathways (Figure 5E). These findings indicate a heightened prevalence of immune responses and cellular proliferation activities mediated by macrophages in DR. The expression levels of immune checkpoints were examined, and a limited number of immune checkpoints, including HLA, LAIR1, CD86, and NRP1, were identified as prominently expressed in macrophages (Figure 5F). Moreover, the analysis of TAM‐related genes revealed subtype‐specific upregulation: CSF1R was elevated in Macro_CD163L1, MARCO and CCL1 were upregulated in Macro_GCHFR, and FCGR3A showed higher expression in Macro_P2RY13 (Figure 5G), indicating distinct functional roles and immunomodulatory potential among the macrophage subpopulations in DR. To elucidate the regulatory role of glycosylation modifications in macrophages, we analyzed the glycosylation scores across five distinct macrophage subtypes. The results indicated that Macro_MIR181A1HG exhibited the highest glycosylation score (Figure 5H). The correlation between each macrophage subtype and the canonical functions of macrophages was investigated. The results showed that Macro_MIR181A1HG exhibited a strong enrichment of M2‐associated gene signatures, while Macro_P2RY13 showed higher enrichment of M1‐associated gene signatures (Figure 5I). In addition, Macro_MALAT1 demonstrated robust associations with angiogenesis and profibrotic functions (Figure 5I). The pathological hallmarks of DR were characterized by aberrant retinal neovascularization and fibroproliferative changes [24, 25], which contribute to vision loss by disrupting retinal architecture and promoting pathological angiogenesis and fibrotic remodeling. To investigate the molecular drivers underlying macrophage differentiation, we incorporated PBMC‐derived monocytes from GSE248284 into the analysis together with monocyte and macrophage populations identified in PDR FVM tissues from GSE245561 and GSE165784. Pseudotime trajectory analysis revealed a progressive transition from circulating monocytes to tissue‐infiltrating monocytes and ultimately to differentiated macrophage subsets. PBMC_Mono was primarily localized at the beginning of the trajectory, whereas Tissue_Mono was distributed throughout the entire differentiation trajectory, and Macro_MIR181A1HG was enriched at the terminal end of the trajectory, indicating that it represents a highly differentiated macrophage subtype and may play a critical role in the PDR microenvironment (Figure 5J). Conclusively, Macro_MIR181A1HG represents a terminally differentiated M2‐like macrophage subtype characterized by high glycosylation activity and enrichment of M2‐associated transcriptional programs, which may act as a key driver of fibrovascular progression in DR.

FIGURE 5Macro_MIR181A1HG represents a proliferative and glycosylation‐active macrophage subtype in PDR. (A) UMAP visualization of 5 cell clusters in macrophage. (B) UMAP plot showing manual annotation of macrophage subtypes. (C) Heatmap depicting the expression of marker genes for different macrophage subtypes. (D) Stacked bar plots illustrating the relative proportion (left) and absolute number (right) of each macrophage subtype across nine PDR samples. (E) GSVA enrichment analysis results for each macrophage subtype. (F) UMAP visualizations of immune‐related regulatory gene expression across macrophages. (G) Bubble plot depicting the expression of genes related to TAMs across different macrophage subtypes. (H) Boxplots showing the distribution of glycosylation scores across different macrophage subtypes. (I) Heatmap of the correlation between macrophage subtypes and canonical macrophage functions. (J) UMAP visualizations of pseudotime analysis results for monocytes and macrophage subtypes using GSE245561, GSE165784, and GSE248284.

**Figure:**
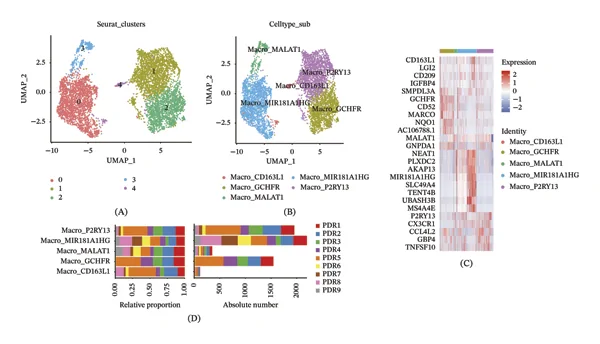

**Figure:**
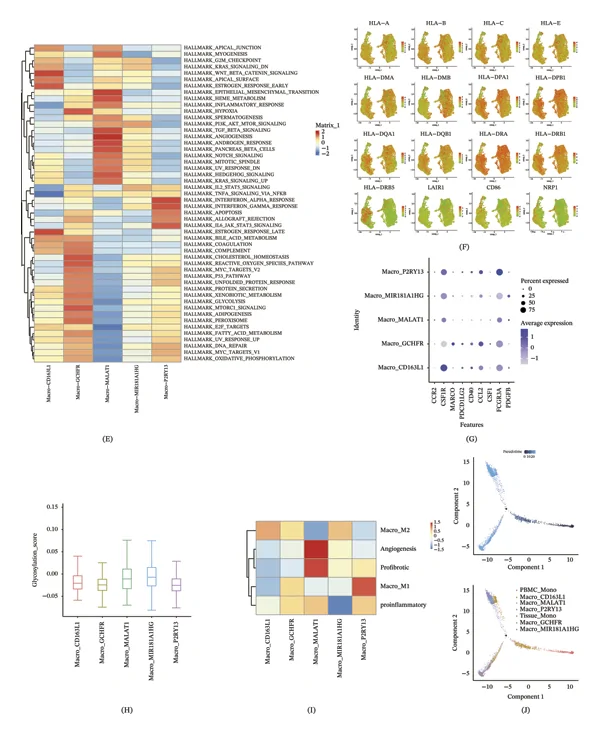

### 3.6. A Total of 862 Glycosylation‐Associated Macrophage Genes Were Identified in DR

Evidence shows a significant link between glycosylation and DR progression [7], underscoring the potential role of glycosylation in modulating cellular function in DR. Given the lack of glycosylation‐associated macrophage diagnostic markers in DR, we aimed to investigate glycosylation‐related states in DR. We analyzed the glycosylation score status of individual cells using ssGSEA, and the results showed that endothelial cells, macrophages, and NK cells exhibited relatively high glycosylation scores (Figure 6A). Cells were stratified into high‐ and low‐glycosylation score groups according to the expression profiles of predefined glycosylation‐related genes (Figure 6B). Compared with the low‐glycosylation group, the high‐glycosylation group exhibited upregulation of ST6GALNAC3, AC093515.1, AC114757.1, AC002070.1, and AC119674.1. Conversely, RPS2P45, RPS13P2, AC116533.1, FCN, and CTGF were more highly expressed in the low‐glycosylation group relative to the high‐glycosylation group (Figure 6B). A total of 1187 DEGs were identified between the high– and low–glycosylation score groups (Figure 6C). Compared to the low–glycosylation score group, 1100 DEGs were upregulated and 87 DEGs exhibited downregulation in the high–glycosylation score group (Figure 6C). The distribution of glycosylation scores across different cells is shown in Figure 6D, highlighting variability among subtypes between the high– and low–glycosylation score group. The box plot illustrated that the glycosylation scores of diverse cell subtypes in the high–glycosylation score group were markedly elevated compared to those in the low–glycosylation score group, indicating a successful stratification strategy based on glycosylation scores (Figure 6E). Among the five macrophage subsets, the Macro_MALAT1 subset displayed the highest glycosylation score (Figure 6F). Moreover, this subset, together with the Macro_MIR181A1HG and Macro_P2RY13 subsets, demonstrated significantly elevated glycosylation scores in the high glycosylation score group compared to the low glycosylation score group (Figure 6G). To explore the regulatory role of glycosylation modification in macrophages, WGCNA analysis was applied to analyze the gene expression modules of macrophages in DR. Eight distinct gene expression modules of macrophages were identified in DR (Figure 6H). Among them, macrophages‐module 3 showed the greatest correlation with glycosylation scores (Figure 6I). We overlapped 1187 DEGs with 4015 macrophages‐M3 genes, a total of 862 glycosylation modification‐related macrophage hub genes were identified (Figure 6J). In summary, a total of 862 glycosylation‐associated macrophage genes were identified in DR.

FIGURE 6A total of 862 glycosylation‐associated macrophage genes were identified in DR. (A) Boxplots showing the distribution of glycosylation scores across different cell subtypes. (B) Dot plot illustrates the expression of glycosylation signature genes in low and high glycosylation score groups. (C) Volcano plot of DEGs between high and low glycosylation score groups. (D) UMAP visualization of cells colored by high (cyan) and low (red) glycosylation score groups. (E) Boxplots comparing glycosylation scores between high and low groups for each cell subtype. (F) Violin plots of glycosylation scores across five macrophage subsets. (G) Boxplots plots of glycosylation scores in the high (cyan) and low (red) glycosylation score groups across five macrophage subsets. (H) Hierarchical clustering dendrogram from hdWGCNA. (I) Correlation heatmap between macrophage modules, glycosylation scores and macrophage type. (J) Venn diagram showing the overlap between 1187 DEGs and 4015 macrophage‐M3 module genes.

**Figure:**
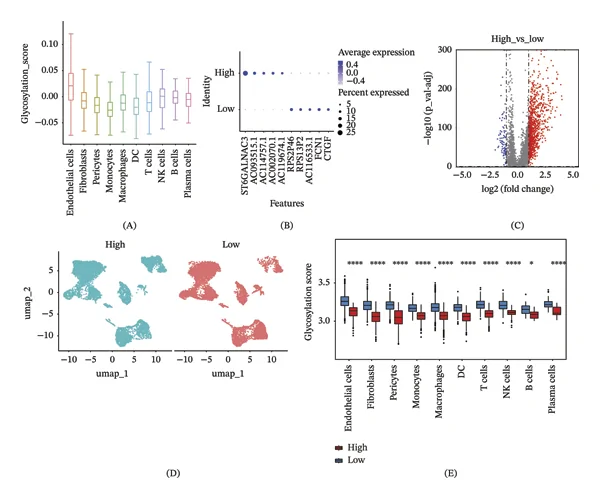

**Figure:**
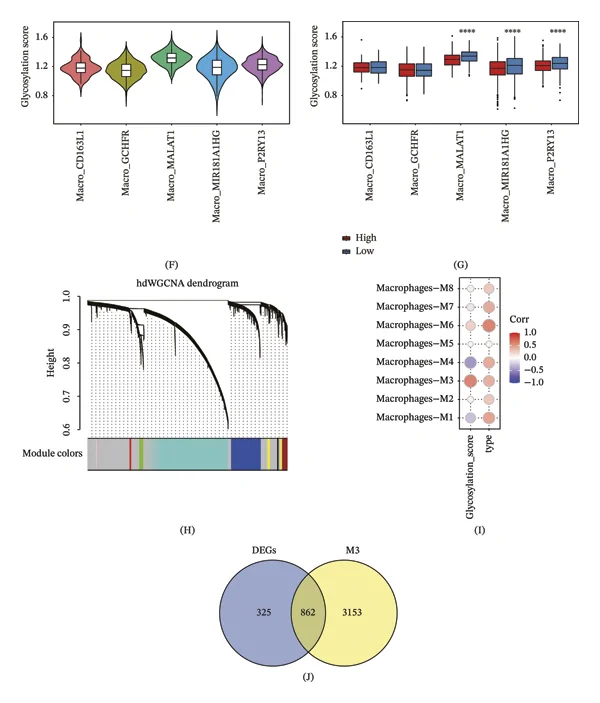

### 3.7. Seven Key MDRGGs Were Identified Through Machine Learning in DR

To identify diagnostic biomarkers, machine learning algorithms were performed to screen the glycosylation‐related macrophage gene of DR. A total of 209 genes were identified from pseudotime‐associated differentiation genes of angiogenic and profibrotic macrophage populations. Upon intersecting 209 genes with 862 glycosylation‐related macrophage hub genes, and 100 MDRGGs were obtained in DR (Figure 7A). Based on 100 MDRGGs, a comprehensive framework was constructed by applying 11 distinct machine learning algorithms in bulk RNA‐seq (GSE102485 and GSE160306). As depicted in Figure 7B, the optimal model combination, glmBoost + Enet [alpha = 0.8], achieved the highest mean AUC score of 0.96. Enet [alpha = 0.8] algorithm identified 14 hub genes in training set GSE160306 (Figure 7C,D), while the glmBoost algorithm specifically pinpointed 11 hub genes (Figure 7E,F). Seven key MDRGGs were determined by integrating the findings from both analytical approaches: AKAP13, SRGAP2, AFF1, ARHGAP24, RNF149, PTK2B, and ATP1B3 (Figure 7G). The diagnostic value of seven hub MDRGGs for DR was tested in the training set (GSE160306). The calculated AUC for each hub MDRGGs was provided for AKAP13 (AUC = 0.665), SRGAP2 (AUC = 0.620), AFF1 (AUC = 0.652), ARHGAP24 (AUC = 0.625), RNF149 (AUC = 0.680), PTK2B (AUC = 0.767), and ATP1B3 (AUC = 0.760) (Figure 7H–N). In summary, the diagnostic model constructed based on the seven MDRGGs has demonstrated robust diagnostic capability in DR.

FIGURE 7Seven key MDRGGs were identified through machine learning in DR. (A) Venn diagram showing the overlap between 862 glycosylation‐associated macrophage genes and 209 monocyte‐related genes. (B) Heatmap showcasing the performance of different ML algorithm combinations in predicting DR. (C) Cross‐validation plot for the Enet model, illustrating the relationship between log(*λ*) and binomial deviance. (D) Coefficient profile plot for the Enet. (E) Cross‐validation plot for the glmBoost model, showing the negative binomial log‐likelihood path across boosting iterations. (F) Coefficient trace plot for the glmBoost model during boosting iterations. (G) Venn diagram depicting the overlap of hub genes identified by Enet (alpha = 0.8) and glmBoost algorithms. ROC curve for the (H) AKAP13, (I) SRGAP2, (J) AFF1, (K) ARHGAP24, (L) RNF149, (M) PTK2B, and (N) ATP1B3 in training set GSE160306.

**Figure:**
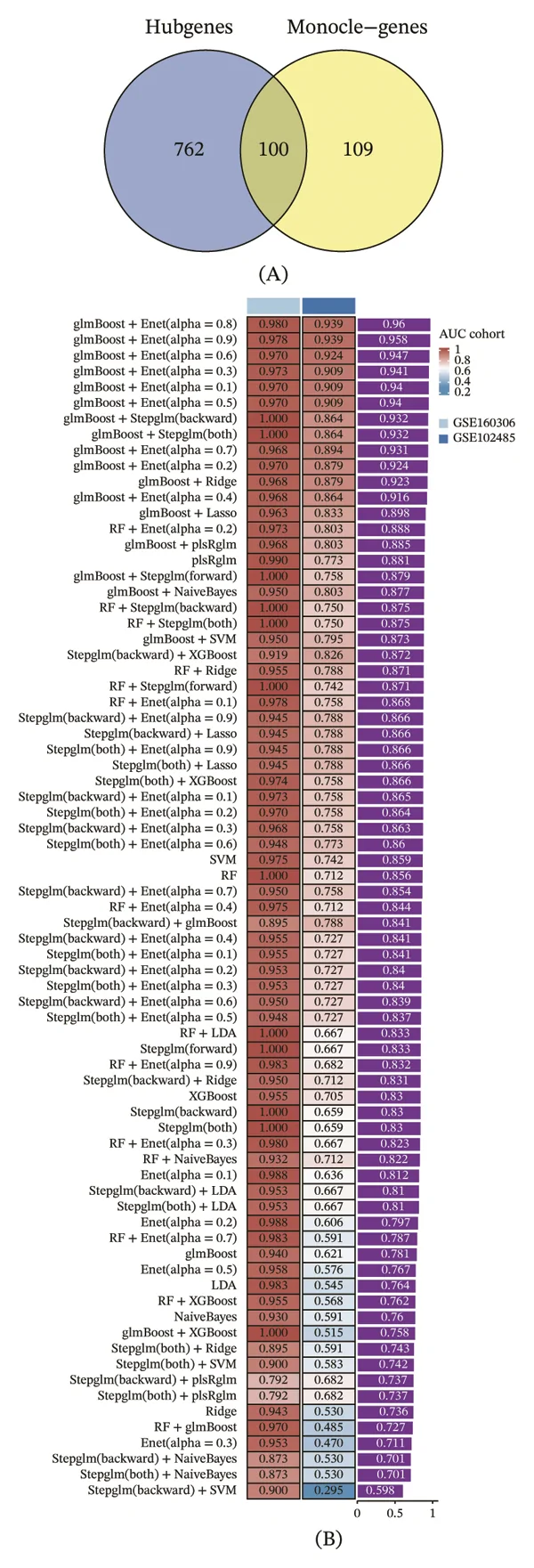

**Figure:**
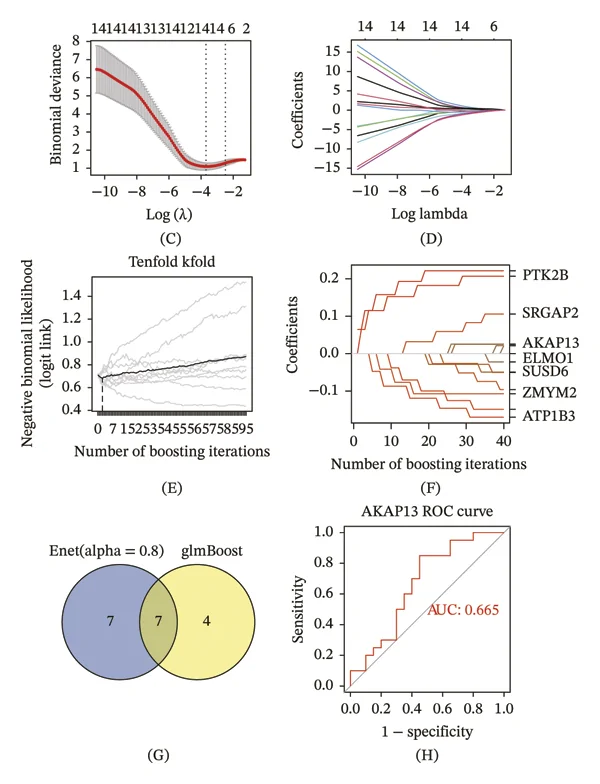

**Figure:**
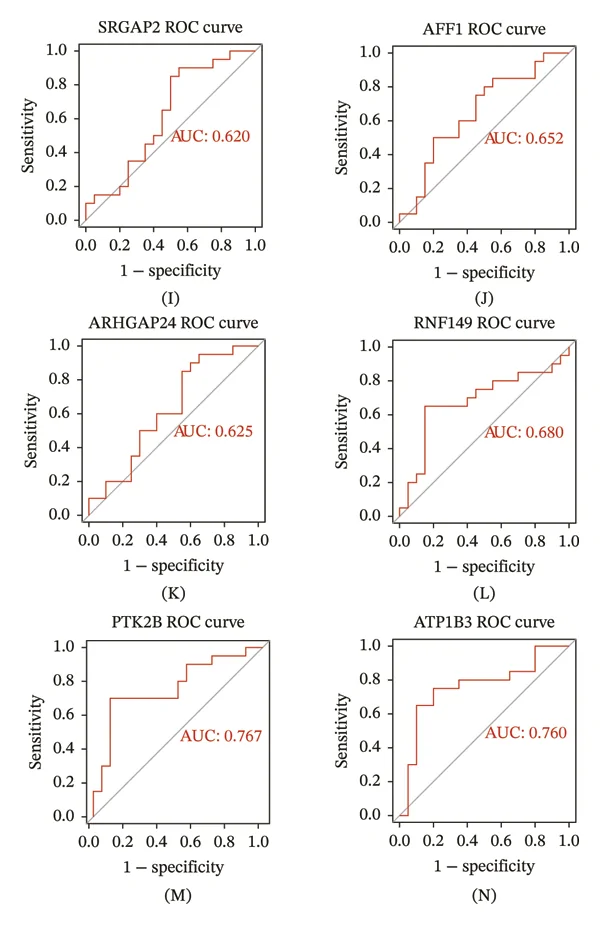

### 3.8. Nomogram of Seven MDRGGs Exhibited Strong Diagnostic Capacity for High‐Risk DR Patients

To further substantiate the diagnostic accuracy of the seven MDRGGs, we assessed the diagnostic performance in the testing cohort (GSE102485). Notably, all seven hug genes achieved AUC values exceeding 0.8, underscoring robust diagnostic capability for DR (Figure 8A–G). A nomogram was constructed in the GSE102485 cohort to improve the diagnostic and predictive performance of the seven MDRGGs (Figure 8H). The calibration curve demonstrated strong concordance between the probabilities predicted by the nomogram‐based diagnostic model and the actual observed outcomes (Figure 8I). In addition, the DCA plot and risk threshold diagram underscored the potential advantages of employing the nomogram in clinical decision‐making processes for the diagnosis for high‐risk individuals of DR (Figure 8J,K). Conclusively, the nomogram of seven MDRGGs exhibited robust clinical diagnostic capacity for high‐risk patients of DR.

FIGURE 8Nomogram of seven MDRGGs exhibited strong diagnostic capacity for high‐risk DR patients ROC curve for the (A) AKAP13, (B) SRGAP2, (C) AFF1, (D) ARHGAP24, (E) RNF149, (F) PTK2B, and (G) ATP1B3 in testing set GSE102485. (H) Nomogram for DR diagnosis displaying the contribution of seven key genes (AKAP13, SRGAP2, AFF1, ARHGAP24, RNF149, PTK2B, ATP1B3) to the total risk score. (I) Calibration curve comparing predicted probabilities and observed probabilities for the nomogram. (J) DCA for the nomogram in the GSE102485 cohort. (K) Risk threshold analysis for the nomogram showing the number of high‐risk individuals and high‐risk individuals with events per 1000 people across cost‐benefit ratios.

**Figure:**
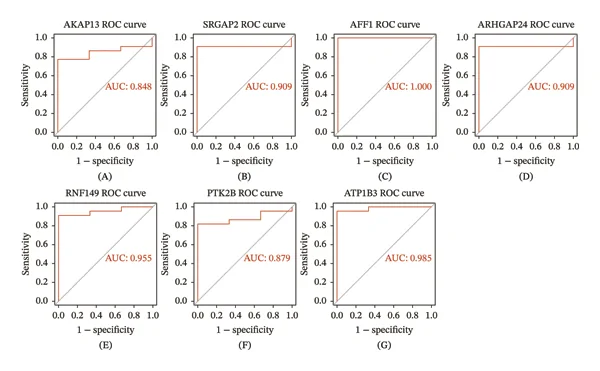

**Figure:**
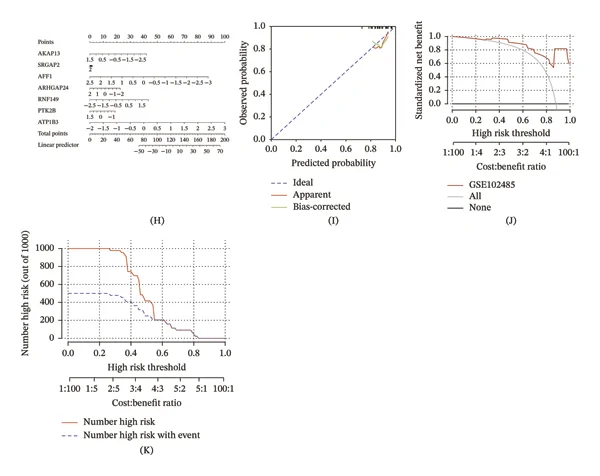

## 4. Discussion

DR remains a leading cause of vision impairment globally, and deciphering its complex pathogenic mechanisms is pivotal for improving clinical diagnosis and intervention. This study leveraged scRNA‐seq data to dissect the role of glycosylation‐regulated macrophage polarization in DR. Meanwhile, machine learning approaches were employed to construct a diagnostic model based on MDRGGs, which exhibited robust diagnostic performance in DR. These results offer a new framework for identifying DR patients, with the potential to inform individualized therapeutic strategies and promote precision medicine in DR care.

Our results observed significant macrophage infiltration and an intricate macrophage–monocyte signaling axis in PDR samples. Investigations have revealed that ischemia and hypoxia in the retina lead to the perivascular accumulation of inflammatory cells such as macrophages and monocytes [26, 27]. Bone marrow‐derived monocytes are recruited to the injured tissue and subsequently differentiate into macrophages in inflammatory contexts [28]. It should be noted that the classical M1/M2 polarization framework represents a simplified model of macrophage activation. Recent single‐cell studies have demonstrated that macrophage states exist along a dynamic continuum with substantial functional plasticity. Therefore, the M1‐and M2‐related signatures used in this study should be interpreted as reference functional programs rather than discrete macrophage identities. For instance, Van Hove et al. identified the presence of inflammatory monocyte‐derived macrophages in the retinas of Akimba mice, which were absent in nondiabetic mice [10]. The infiltrating macrophages drive chronic inflammation and consequently lead to delayed tissue repair in diabetic settings by secreting proinflammatory cytokines [29]. In addition to proinflammatory functions, monocyte‐derived macrophages can also alternatively initiate the resolution of inflammation to facilitate tissue repair [3]. This study showed that macrophage demonstrated the highest outgoing interaction strength via the SPP1 pathway. MKI67 microglia interacts with endothelial cells via the SPP1‐ITGA4 signaling pathway, thereby promoting angiogenesis in PDR [30]. In diabetic kidney disease (DKD), SPP1 facilitates macrophage infiltration and interstitial fibrosis, thereby driving disease progression [31]. These studies may indicate that macrophages potentially engage in angiogenesis and profibrotic processes in DR via SPP1. Importantly, our pseudotime analysis suggested a progressive transition from circulating monocytes toward distinct macrophage states, indicating that macrophage differentiation may represent a dynamic component of the PDR microenvironment. Combined with the prominent SPP1‐mediated intercellular communication identified in this study, these findings support a model in which differentiated macrophage populations actively participate in retinal neovascularization and fibrovascular remodeling.

Our exploration revealed that the elevated glycosylation scores in macrophages, endothelial cells, and T cells suggest coordinated post‐translational reprogramming during DR progression. Glycosylation, as a post‐translational modification, is significantly associated with the generation of a proinflammatory microenvironment, vascular dysfunction, and disease progression in DR [7, 8, 32]. Macrophage subclustering revealed that Macro_MIR181A1HG exhibited both the highest glycosylation score and strongest M2 macrophage polarization. This was consistent with the literature. The O‐GlcNAc glycosylation level and O‐GlcNAc transferase activity are elevated to modify the Thr717 site of STAT3, thereby activating the IL‐6/STAT3 signaling pathway and promoting the M2 polarization of macrophages [33]. Pseudotime trajectory analysis further established Macro_MIR181A1HG as the terminal differentiation state originating from PBMC‐derived monocytes. Notably, Macro_MIR181A1HG displayed both the highest glycosylation activity and the strongest enrichment of reparative macrophage‐related programs, suggesting that glycosylation‐associated transcriptional reprogramming may accompany macrophage maturation and functional specialization during DR progression. Rather than representing a fixed macrophage phenotype, this population may reflect a differentiated cellular state shaped by the diabetic inflammatory microenvironment. The terminal differentiation of PBMC‐derived monocytes into macrophages is influenced by glycosylation. Glycosylation‐engineered nanoparticles, such as IL10‐GE‐PNPs, have been shown to regulate macrophage polarization in vivo, promoting an anti‐inflammatory M2 phenotype and enhancing tissue repair [34]. Additionally, O‐GlcNAcylation of proteins has been shown to impair cellular functions in diabetic contexts, highlighting the broader impact of glycosylation on protein‐mediated regulatory networks. The macrophage M3 module was strongly correlated with glycosylation scores and differentiation, implying that glycosylation modifications critically correlated macrophage polarization in DR. These findings showed that glycosylation played a pivotal role in shaping macrophage function and polarization in DR.

The diagnostic model incorporating seven MDRGGs (AKAP13, SRGAP2, AFF1, ARHGAP24, RNF149, PTK2B, and ATP1B3) achieved exceptional performance in DR, while the nomogram provided clinically actionable risk stratification. Several of these genes have been implicated in biological processes relevant to DR pathogenesis. AKAP13 has been identified as a regulator of TLR2 signaling, a pathway involved in retinal inflammation and microglia activation during DR progression [35, 36]. SRGAP2 promotes retinal ganglion cell degeneration in mice [37], suggesting a potential link to the neurodegenerative component of DR. AFF1 regulates endothelial cell function through the PI3K/AKT pathway in gestational DM [38], whereas ARHGAP22, a member of the same Rho GTPase‐activating protein family as ARHGAP24, has been associated with elevated circulating endothelial progenitor cells in patients with DR [39]. These findings suggest that the identified genes may participate in inflammatory responses, neuroretinal injury, and vascular dysfunction, which are key pathological features of DR. In addition, PTK2B has been reported as a promising biomarker for predicting the progression of diabetic neuropathy [40], while ATP1B3 is associated with chronic inflammation in Type 2 diabetes [41]. Among the seven MDRGGs, RNF149 remains the least characterized in the context of DR. To our knowledge, no study has directly linked RNF149 to DR progression, highlighting it as a potentially novel candidate for future investigation. Given that endothelial dysfunction, neuroretinal degeneration, chronic inflammation, and immune remodeling are central mechanisms underlying DR progression [42, 43], the identified MDRGGs may serve not only as diagnostic biomarkers but also as candidate molecular mediators linking glycosylation‐associated macrophage dysregulation to disease development.

While our integrated approach provides unprecedented resolution in delineating macrophage heterogeneity and glycosylation‐related transcriptional programs in DR, several limitations should be acknowledged. First, the scRNA‐seq datasets included a relatively limited number of PDR samples, and the diagnostic model was developed using independent bulk RNA‐seq cohorts. Differences in sample size, disease stage, and tissue composition may affect the generalizability of our findings. Therefore, validation in larger, stage‐matched cohorts is warranted. Second, the analyzed single‐cell datasets were primarily derived from PDR FVM, where resident retinal microglia and infiltrating monocyte‐derived macrophages may exhibit overlapping transcriptional profiles. Although the macro‐P2RY13 subset expressed microglia‐associated markers such as P2RY13 and CX3CR1, the lack of lineage‐tracing information and definitive microglial markers prevented a rigorous distinction between these populations. Finally, the biological functions of the seven MDRGGs, particularly their glycosylation‐dependent regulation, require further experimental validation. Future studies exploring the therapeutic potential of targeting macrophage subtypes or glycosylation pathways may provide additional insights into DR pathogenesis and treatment strategies.

## 5. Conclusion

In conclusion, our study deciphered the critical role of glycosylation‐related macrophage polarization in DR at single‐cell resolution and translated the discovery into a clinically applicable diagnostic model. These findings not only advance our understanding of DR biology but also pave the way for personalized diagnosis and targeted therapies, ultimately aiming to improve outcomes for patients with this devastating disease.

**Nomenclature**
DCA: Decision curve analysis
DEGs: Differentially expressed genes
DKD: Diabetic kidney disease
DM: Diabetes mellitus
DR: Diabetic retinopathy
FVM: Fibrovascular membrane
Enet: Elastic net
GEO: Gene Expression Omnibus
glmBoost: Generalized linear model boosting
k‐NN: k‐nearest neighbor
LASSO: Least absolute shrinkage and selection operator
LDA: Linear discriminant analysis
MDRGGs: Macrophage differentiation–related glycosylation genes
NaiveBayes: Naive Bayes classifier
PBMCs: Peripheral blood mononuclear cells
PCA: Principal component analysis
PDR: Proliferative diabetic retinopathy
plsRglm: Partial least squares regression for GLM
RF: Random forest
Ridge: Ridge regression
ROC: Receiver operating characteristic
scRNA‐seq: Single‐cell RNA sequencing
ssGSEA: Single sample gene set enrichment analysis
Stepglm: Stepwise generalized linear model
SVM: Support vector machine
UMAP: Uniform manifold approximation and projection
WGCNA: Weighted gene coexpression network analysis
XGBoost: Extreme gradient boosting

## Funding

This study was supported by the Medical Scientific Research Foundation of Guangdong Province, China (No. B2023448) and Key Laboratory of Guangdong Higher Education Institutes (No. 2021KSYS009).

## Conflicts of Interest

The authors declare no conflicts of interest.

## Supporting Information

Additional supporting information can be found online in the Supporting Information section.

## Supporting information


**Supporting Information** Figure S1. Quality control and overview of single‐cell RNA‐seq datasets in DR. (A) Violin plots showing the distributions of nFeature_RNA, nCount_RNA, and percent mitochondrial gene expression (percent.mt) across all cells before filtering. (B) Scatter plots illustrating the relationships between nCount_RNA and percent.mt, and between nCount_RNA and nFeature_RNA, respectively. The observed weak negative correlation between nCount_RNA and percent.mt and strong positive correlation between nCount_RNA and nFeature_RNA indicate overall high data quality and effective removal of low‐quality cells.

## Data Availability

The datasets used and analyzed during the current study are available from the publicly available GEO database: GSE245561, GSE165784, GSE248284, GSE102485, and GSE160306. The expression data supporting this study are from previously reported databases, which have been cited. The data that support the findings of this study are available anonymously from the corresponding author. Further inquiries can be directed at the corresponding author.
