# Supplementary material for: Glycosylation‐Associated Macrophage Signatures Define a Diagnostic Model for Diabetic Retinopathy via Single‐Cell Analysis
Source: J Ophthalmol. 2026 Aug 27;2026:7199483. doi: 10.1155/joph/7199483 (PMC13520116; doi:10.1155/joph/7199483)
Supplement: Supplementary file 1 [file JOPH-2026-7199483-s001.zip › Figure S1.pdf]

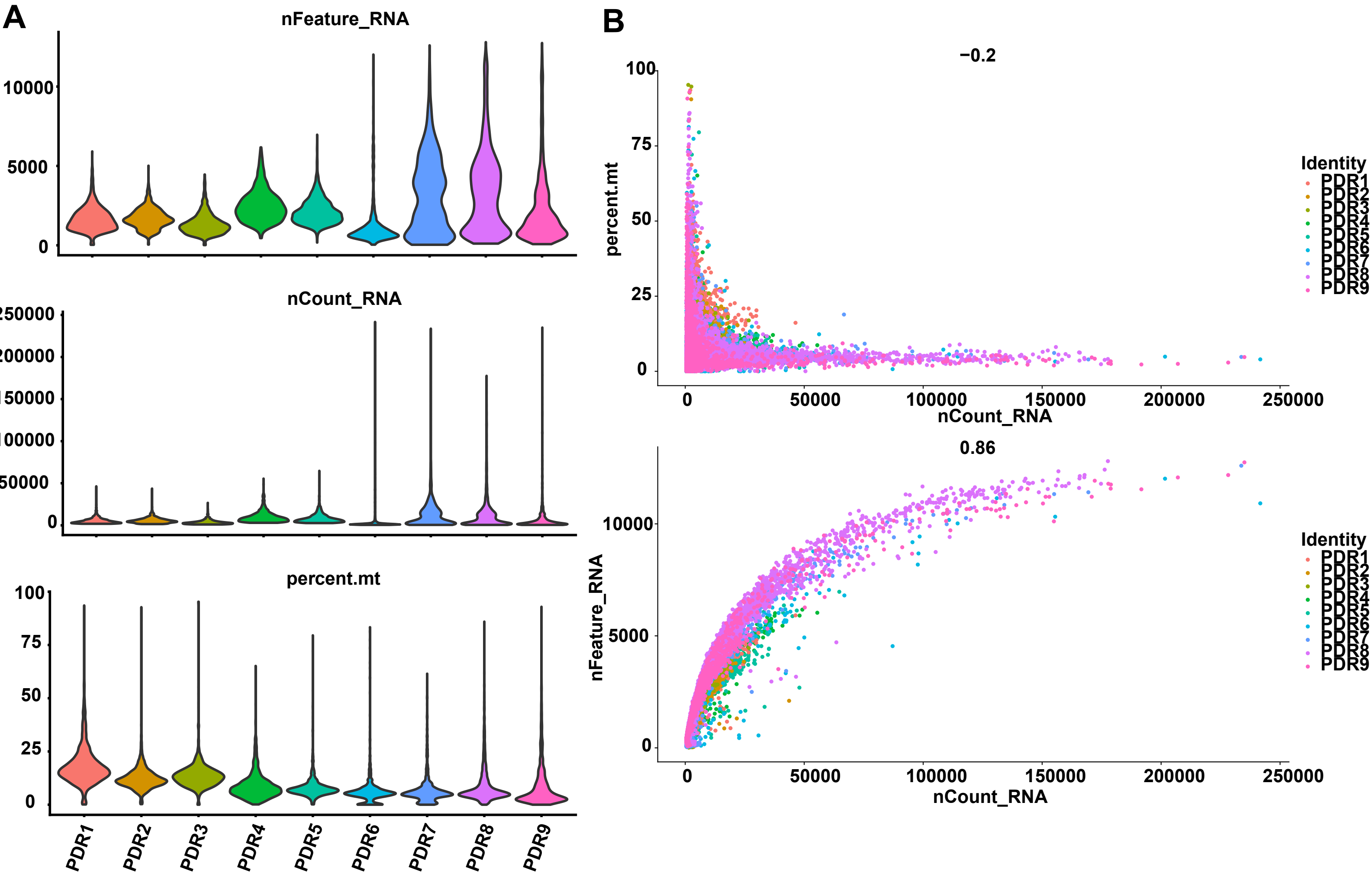
A
nFeature_RNA
10000
5000
0
nCount_RNA
250000
200000
150000
100000
50000
0
percent.mt
100
75
50
25
0
PDR1
PDR2
PDR3
PDR4
PDR5
PDR6
PDR7
PDR8
PDR9
B
−0.2
percent.mt
nCount_RNA
0
50000
100000
150000
200000
250000
Identity
PDR1
PDR2
PDR3
PDR4
PDR5
PDR6
PDR7
PDR8
PDR9
0.86
nFeature_RNA
nCount_RNA
